# Inflammatory Biomarkers and Carotid Thickness in HIV Infected Patients under Antiretroviral Therapy, Undetectable HIV-1 Viral Load, and Low Cardiovascular Risk

**DOI:** 10.5935/abc.20190230

**Published:** 2020-01

**Authors:** Kaliene Maria Estevão Leite, Gerson Gomes Santos Júnior, Emmanuelle T. A. M Godoi, Adriana Ferraz Vasconcelos, Virgínia Maria Barros Lorena, Paulo Sérgio Ramos Araújo, Kledoaldo Oliveira Lima, Heloisa Ramos Lacerda

**Affiliations:** 1 Universidade Federal de Pernambuco - Pós-Graduação em Medicina Tropical, Recife, PE - Brazil; 2 Instituto de Pesquisa Aggeu Magalhães, Recife, PE - Brazil

**Keywords:** HIV, HIV Infections, Cardiovascular Diseases, Antiretroviral Therapy, Risk Factors, Caroti Artery Diseases, Atherosclerosis

## Abstract

**Background:**

People living with HIV are at increased risk of cardiovascular disease and carotid thickness, due to the inflammation caused by the virus, the antiretroviral therapy, and other risk factors. However, few studies have observed the occurrence of cardiovascular diseases and carotid thickness in HIV-positive population at low cardiovascular risk and with undetectable viral load.

**Objectives:**

To evaluate the association between levels of inflammatory markers and carotid thickness in people living with HIV, under antiretroviral therapy and at low cardiovascular risk.

**Methods:**

To determine low cardiovascular risk in both groups (HIV infected and non-infected individuals), the Framingham Risk Score was used. Inflammatory markers (IFN-γ, TNF-α, IL-1β, IL-6, sVCAM-1, and sICAM-1) were assessed using flow cytometry. Carotid thickness (mm) was measured using Doppler ultrasound. Level of significance was p < 0.05.

**Results:**

In People living with HIV, age and smoking status were associated with carotid thickness alterations. In the non-HIV group, age, higher total cholesterol, and LDL levels were associated with increased carotid thickness. Using the multivariate analysis, a significant association between TNF-α and IL- 1( levels, and a higher chance of atherosclerosis development in HIV group were observed.

**Conclusions:**

Both groups have a similar risk for developing cardiovascular disease, therefore our study demonstrates that HIV-positive individuals with undetectable viral load in antiretroviral therapy without protease inhibitors and with low cardiovascular risk do not present differences in carotid thickness in relation to uninfected individuals.

## Introduction

Increased longevity in people living with HIV (PLHIV) due to effective highly active antiretroviral therapy (HAART) has increased the incidence of chronic diseases, such as cardiovascular disease.^1^ According to some studies, the virus and antiretroviral therapy (ART) are factors that favor the increase of inflammatory makers and carotid thickness.^2,3^ HIV-infected individuals have high levels of C-reactive protein, which is associated with atherosclerosis and myocardial infarction. Levels of interleukin-6 (IL-6), tumour necrosis factor alpha (TNF-α), interferon gamma (IFN-γ), interleukin-1 (IL-1), intracellular cell adhesion molecule (sICAM), and vascular cell adhesion molecule (sVCAM), which rise in the progression of cardiovascular disease, are also increased in this population.^4-6^

Studies did not evaluate factors of cardiovascular disease progression in the population considered to be at low cardiovascular risk, without observing the true possible effect of the inflammation caused by the virus and the adverse effect of antiretrovirals without the interference of intrinsic factors affecting cardiovascular risk in these individuals. In addition, we have not yet studied a population in which all patients had undetectable HIV-1 RNA viral load and used only nucleoside reverse transcriptase inhibitor (NRTIs) analogues and non-nucleoside reverse transcriptase inhibitors (NNRTIs), since protease inhibitors (PIs) have high adverse effect on cardiovascular disorders.

The present study evaluated the association of inflammatory markers IFN-γ, IL-1β, IL-6, TNF-α, C-reactive protein, sVCAM-1, and sICAM-1 and carotid thickness in HIV infected peope, in use of NRTIs analogues and NNRTIs, and low cardiovascular risk. In addition, carotid intima-media thickness and inflammatory markers levels between HIV-infected and non-infected individuals stratified by age were compared.

## Method

### Study subjects

Cross-sectional analytical study with 115 patients was conducted at *Hospital das Clínicas* - Federal University of Pernambuco, Northeast, Brazil. Individuals were enrolled by convenience sampling. Ninety-nine patients were infected with HIV (HIV group) and were attended at the Specialized HIV/AIDS Healthcare Service, other 16 individuals were healthy and used as control (non-HIV group); both groups were aged between ≥ 18 and ≤ 60 years. All HIV patients were under ART with two NRTIs analogues and one NNRTI started at any time from their diagnosis, had undetectable HIV-1 RNA viral load, and were not on therapy for dyslipidaemia. Healthy controls were followers of patients attending in the Urology Service of the same hospital. Low risk for cardiovascular disease was also an inclusion criterion for both groups, calculated by the Framingham Risk Score (FRS). FRS estimates the likelihood of myocardial infarction or death from coronary disease within 10 years in individuals without prior clinical atherosclerosis. Risk calculation uses parameters such as gender, age, total and HDL cholesterol levels, systolic blood pressure, and smoking status.^7^

### Data collection

After patients signed the informed consent form, data were collected with standardized questionnaires, based on medical records and/or interview information as follows: age, gender, race, ART type and time, CD4+ T cells count, HIV-1 RNA viral load, and smoking and diabetes status. CD4+ T-cell counts were estimated with flow cytometry using the FACSCalibur (Becton-Dickinson, USA) and results were expressed in cells/mm^3^. HIV viral load was measured using real-time polymerase chain reaction (RT-PCR) (Roche Diagnostics, Germany) with detection limit of 50 copies/mL. Afterwards, the examinations of lipidogram, the measurements of carotid intima-media thickness (CIMT), and the assessment of inflammatory biomarker levels were carried out. The moment the patient was included in the study, blood was collected for lipidogram and inflammatory biomarker determinations. Blood pressure assessment and carotid Doppler ultrasound were performed as well.

### Lipidogram

Total cholesterol, HDL, and triglycerides were examined using the automated analyser CMD800i (Wiener LAB) with photometric methodology. Blood was collected without anticoagulant and was immediately sent to the laboratory for analysis. LDL and VLDL cholesterol values were obtained through the Friedwald formula.

### Inflammatory markers

Inflammatory markers (IFN-γ, TNF-α, IL-1β, IL-6, sVCAM-1, and sICAM-1) were assessed using the cytometric bead array (CBA) method. Results were generated in tabular and graphical format using the BD CBA Software FCAP Array, version 3.01. Ultrasensitive C-reactive protein was measured through the latex immunoblottomymetry technique using the CMD800i automated analyzer (Wiener LAB), where it reacts with the specific antibody to form insoluble immunocomplexes. The turbidity produced by immunocomplexes is proportional to the PCR concentration in the sample.

### Measurements of the carotid intima-media thickness

Measurement was performed using an ultrasound device (General Eletric, model LOGIQe BT12), which features DICOM 3.0 software and Auto IMT, with automatic and well-monitored images. Imaging exams were performed by two medical vascular surgeons. Measurements were performed on the posterior wall of the studied vessel in a plateau-free area and defined as the distance between two echogenic lines represented by the lumen-intima and media adventitia interface of the arterial wall. The mean automatic measurement of the thickened common carotid artery was defined as either right (RCC) or left (LCC). Presence of plaque was considered when intima-media thickening (IMT) > 1.5 mm was observed.^8-10^

### Statistical analysis

Statistical analyses were performed using the STATA software version 11.0. Level of significance was p < 0.05. Variables were also analyzed stratified by age, with cutoff point at 40 years due to the distribution of N in the cases group. Qualitative variables were expressed through absolute and relative frequencies, and the quantitative ones, through descriptive statistics, such as mean, standard deviation, median, 25^th^ and 75^th^ percentile. Continuous variables that presented normal distribution were described through mean and standard deviation; in case of non-normal distribution, they were described through median and interquartile range. Normal distribution was observed for quantitative variables age, total cholesterol, HDL and LDL, while triglycerides, CIMT and inflammatory markers did not present normal distribution. Data normality was evaluated by the Kolmogorov-Smirnov test. Nonparametric Kruskal-Wallis and Mann-Whitney tests were used to compare medians. Student’s independent *t*-test was used to compare means between groups (HIV^+^ and HIV^-^). Correlation analysis was performed using the Spearman coefficient. To select the most significant variables, a stepwise logistic regression was used. Variables moderately associated (p < 0.2) with the dependent variable were included in the model, whereas a threshold of p < 0.05 was adopted for the stepwise elimination of variables considered as risk factors.

### Ethical considerations

The Ethics Committee of the Health Sciences Center of the Federal University of Pernambuco (No. 307087) approved this research and the study protocol conforms to the ethical guidelines of the 1975 Declaration of Helsink. All subjects signed an informed consent form.

## Results

Ninety-nine HIV-positive and 16 non-infected individuals participated in the study. Individuals aged 40 years old or older accounted for 59.6% of those infected with HIV and 75% of the control group. Comparing the characteristics of the groups, there were differences in inflammatory markers IFN-γ, IL-1, and TNF-α, with higher levels in the control group. Regarding CIMT, there was no significant difference between the groups, and distribution of gender as well as smoking and diabetes status were similar among them. The following two variables had borderline significance: levels of HDL cholesterol were higher among patients with HIV, while mean LDL was lower in this group. In the HIV-positive group, 37% of patients had less than five years of ART, 29.3% between 5 and 10 years, and 33.7% more than 10 years. Regarding CD4 levels and viral load during study admission, 90.5% of the subjects had CD4 levels above 350cells/mm^3^ and all had undetectable viral load. Among the therapeutic regimens with NRTIs analogues, 98 (98.98%) of them contained lamivudine, 65 (65.65%) contained zidovudine, 32 (32.32%) contained tenofovir, and three (3.03%) contained didanosine. For NNRTIs, 93 (93.93%) used efavirenz and six (6.06%) used nevirapine (Table 1).

**Table 1:** Comparison between groups of HIV-infected and non-infected patients regarding demographic and clinical characteristics, risk factors for cardiovascular disease, inflammatory markers, carotid intima-media thickness measures, and risk factors for infection

Variables	Groups		p-value
	HIV+ (n = 99)	HIV- (n = 16)	
Agea (years)	42.2 ± 8.9	48.7 ± 9.1	0.007
**Age group**			
< 40 years old	40 (40.4%)	4 (25.0%)	0.239
40 years old or older	59 (59.6%)	12 (75.0%)	
**Gender**			
Female	39 (39.4%)	4 (25.0%)	0.404
Male	60 (60.6%)	12 (75.0%)	
**Smoking**			
Yes	12 (12.1%)	0 (-)	0.213
No	87 (87.9%)	16 (100%)	
**Diabetes**			
Yes	3 (3.0%)	0 (-)	1.000
No	96 (97.0%)	16 (100%)	
**Lipidogram (mg/dL)**			
Total Cholesterol*	184.0 ± 35.4	190.37 ± 48.9	0.528
HDL*	50.3 ± 14.2	43.6 ± 7.8	0.069
LDL*	105.3 ± 27.2	118.5 ± 38.2	0.093
Triglycerides^†^	116.2 (79.6; 176)	120.1 (90.1; 191.3)	0.695
**CIMT (mm)**			
CIMT means*	0.573 ± 0.123	0.586 ± 0.116	0.697
**Inflammatory markers^†^**			
PCR US	0.1 (0; 0.4)	0.1 (0; 0.3)	0.680
ICAM-1	0 (0; 0)	0 (0; 0)	1.000
VCAM-1(x10^-3^)	12.12 (11.42; 12.62)	12.94 (10.59; 13.47)	0.071
IFN	2.16 (1.98; 2.40)	2.67 (2.29; 2.91)	0.002
IL-1	2.87 (2.87; 2.87)^‡^	2.87 (2.87; 4.08)	0.027
IL-6	2.1 (2.1; 2.1)^‡^	2.1 (2.1; 2.1)^‡^	0.689
TNF-α	2.26 (2.26; 2.26)^‡^	2.26 (2.26; 7.55)	0.005
**Time of ART (years)**			
< 5 years	34 (37.0%)	-	-
> 5 and < 10 years	27 (29.3%)	-	
≥ 10 years	31 (33.7%)	-	
**CD4+ Cells Count (Cells/mm^3^)**			
< 200	2 (2.1%)	-	-
200-349	7 (7.4%)	-	
≥ 350	86 (90.5%)	-	
**Antiretrovirals**			
Lamivudine	98 (98.98%)		
Zidovudine	65 (65.65%)		
Tenofovir	32 (32.32%)		
Didanosine	3 (3.03 %)		
Efavirenz	93 (93.93%)		
Nevirapine	6 (6.06%)		

* Mean ± SD - Independent Student's t-test was applied.

† Median (P25; P75) - Kruskall-Wallis test was applied.

‡ Values below the detection limit of the test.

HDL: high-density lipoprotein; LDL: low-density lipoprotein; CIMT: carotid intima-media thickness; ART: antiretroviral therapy.

In HIV-infected individuals, higher HDL and LDL levels, CIMT, CD4+ T cell counts, and ART time were observed in older individuals (Table 2). The 75^th^ percentile calculated for 115 patients was 0.61 mm. Therefore, the CIM was considered thickened if > 0.61 mm. In the HIV-positive group, CIMT ≥ 0.61 mm was detected in 51 individuals (51.51%), of whom 78.4% were aged 40 years old or older. For the non-HIV group, the presence of IMT ≥ 0.61 mm was 56.25% (nine subjects), and 88.9% of these patients were 40 years old or older. Although it was evidenced that HIV-infected individuals were aged 40 years old or older were associated with increased carotid thickness, a comparison between crude and Mantel-Haenszel odds ratios showed that the association between older age and thickness is independent of the infection status. Higher levels of total and LDL cholesterol were associated with CIMT ≥ 0.61 mm in the non-HIV group (Table 3). In the multivariate analysis, after adjustments for age, smoking status, and cholesterol level were made, a significant association with TNF-α levels was observed. Thus, increased levels of TNF-α were associated with greater chance of atherosclerosis. Individuals with increased IL1-β levels had greater chance of atherosclerosis with p-value close to significance (Table 4).

**Table 2:** Comparison of demographic and clinical, laboratory, mean carotid intima-media thickness, and age-related antiretroviral therapy characteristics of HIV-infected individuals

Variables	Age group		p-value
	< 40 years old (n = 40)	≥ 40 years old (n = 59)	
**Gender**			
Female	14 (35.0%)	25 (42.4%)	0.461
Male	26 (65.0%)	34 (57.6%)	
**Smoking**			
Yes	3 (7.5%)	9 (15.2%)	0.246
No	37 (92.5%)	50 (84.8%)	
**Diabetes**			
Yes	1 (2.5%)	2 (3.4%)	0.800
No	39 (97.5%)	57 (96.6%)	
**Lipidogram (mg/dL)**			
Total cholesterol*	169.3 ± 33.1	193.9 ± 33.8	0.001
HDL*	45.6 ± 9.9	53.4 ± 15.8	0.007
LDL*	97.5 ± 23.4	110.9 ± 28.5	0.002
Triglycerides^†^	130.5 (76; 199)	111.4 (81; 174)	0.571
CIMT (mm)*			
CIMT mean	0.521 ± 0.070	0.609 ± 0.138	< 0.001
**Inflammatory markers^†^**			
PCR US	0.2 (0; 0.4)	0.1 (0; 0.3)	0.298
ICAM-1	0 (0; 0)	0 (0; 0)	1.000
VCAM-1(x10^-3^)	12.06 (11.59; 12.49)	12.12 (10.42; 12.75)	0.764
IFN	2.18 (2.05; 2.42)	2.16 (1.91; 2.37)	0.255
IL-1	2.87 (2.87; 2.87)	2.87 (2.87; 2.87)	0.529
IL-6	2.1 (2.1; 2.1)	2.1 (2.1; 2.1)	0.689
TNF-α	2.26 (2.26; 2.26)	2.26 (2.26; 2.26)	0.522
**Time of ART (years)**			
< 5	20 (54.1%)	14 (25.5%)	0.005
5-10	11 (29.7%)	16 (29.1%)	
≥ 10	6 (16.2%)	25 (45.4%)	
**CD4+ T Cells Count (Cells/mm^3^)**			
< 200	0 (2.1%)	2 (3.5%)	0.013
200-349	6 (7.4%)	1 (1.8%)	
≥ 350	32 (84.2%)	54 (94.7%)	

* Mean ± SD - Independent Student's t-test was applied.

† Median (P25; P75) - Kruskall-Wallis test was applied.

HDL: high-density lipoprotein; LDL: low-density lipoprotein; CIMT: carotid intima-media thickness; ART: antiretroviral therapy

**Table 3:** Risk factors related to carotid intima-media thickness, stratified according to the condition of HIV infection

Variables	HIV+			HIV-		
	< 0.61 mm	≥ 0.61 mm	p-value	< 0.61 mm	≥ 0.61 mm	p-value
Age^a^ (years)	37.9 ± 7.1	46.3 ± 8.5	< 0.001	44.0 ± 9.8	52.4 ± 7.0	0.063
**Age group**						
< 40	29 (60.4%)	11 (21.6%)	< 0.001^b^	3 (42.9%)	1 (11.1%)	0.192^†^
≥ 40	19 (39.6%)	40 (78.4%)		4 (57.1%)	8 (88.9%)	
**Gender**						
Female	19 (39.6%)	20 (39.2%)	0.970	2 (28.6%)	2 (22.2%)	0.608
Male	29 (60.4%)	31 (60.8%)		5 (71.4%)	7 (77.8%)	
**Smoking**						
Yes	3 (6.3%)	9 (17.7%)	0.082	0 (-)	0 (-)	-
No	45 (93.7%)	42 (82.3%)		7 (100%)	9 (100%)	
**Diabetes**						
Yes	1 (2.1%)	2 (3.9%)	0.594	0 (-)	0 (-)	-
No	47 (97.9%)	49 (96.1%)		7 (100%)	9 (100%)	
**Lipidogram (mg/dL)**						
Total cholesterol*	178.1 ± 33.8	199.9 ± 58.3	0.395	175.7 ± 31.5	191.8 ± 37.4	0.023
HDL*	41.5 ± 9.8	45.2 ± 5.9	0.362	50.4 ± 17.6	50.1 ± 10.3	0.931
LDL*	110.9 ± 23.3	124.4 ± 47.4	0.504	98.5 ± 21.7	111.4 ± 30.2	0.020
Triglycerides^‡^	113 (72; 181)	118 (86; 174)	0.459	117 (86; 169)	122 (101; 200)	0.560
**Inflammatory markers^‡^**						
PCR US	0.1 (0; 0.4)	0.1 (0; 0.4)	0.966	0.1 (0; 0.6)	0.1 (0; 0.1)	0.581
ICAM-1^¥^	-	-	-	-	-	-
VCAM-1(x10^-3^)	12.1 (11.3; 12.6)	12.0 (11.4; 12.6)	0.931	13.0 (6.6; 13.6)	12.9 (10.8; 13.4)	1.000
IFN	2.16 (1.98; 2.37)	2.21 (1.93; 2.43)	0.481	2.91 (1.96; 3.24)	2.66 (2.32; 2.72)	0.368
IL-1	2.87 (2.87; 2.87)	2.87 (2.87; 2.87)	1.000	2.87 (2.87; 4.40)	2.87 (2.87; 3.77)	0.597
IL-6^¥^	-	-	-	-	-	0.149
TNF-α	2.26 (2.26; 2.26)	2.26 (2.26; 2.26)	0.328	2.26 (2.26; 6.75)	2.26 (2.26; 13.7)	0.634
**Time of ART (years)**						
≤ 5	19 (43.2%)	15 (31.2%)	0.383	-	-	-
5-10	13 (29.5%)	14 (29.2%)		-	-	-
≥ 10	12 (27.3%)	19 (39.6%)		-	-	-

* Media ± standard deviation - Independent Student's t-test was applied.

† OR Crude, 5.47 (2.41-12.93); OR Mantel-Haenszel, 5.60 (2.43-12.9).

‡ Median (P25; P75) - Mann-Whitney test.

¥ There is no variation (all values equal to the minimum).

HDL: high-density lipoprotein; LDL: low-density lipoprotein; ART: antiretroviral therapy.

**Table 4:** Association of factors risks related to carotid intima-media thickness, multivariate analysis stratified according to HIV infection status

Inflammatory markers^‡^	HIV+		HIV-	
	CIMT > 0.61 mm OR (95%CI)*	p-value	CIMT > 0.61 mm OR (95%CI)^†^	p-value
PCR US	1.17 (0.45-2.98)	0.747	0.31 (0.02-5.31)	0.425
VCAM-1(x10^-3^)	0.54 (0.21-1.38)	0.197	1.08 (0.07-17.4)	0.954
IFN	1. 76 (0.69-4.51)	0.238	-	-
IL-1	10.4 (0.71-151.2)	0.087	6.14 (0.24-156)	0.272
TNF-α	31.2 (2.70-361)	0.006	3.06 (0.12-79.3)	0.500

* Adjusted by age and smoking.

† Adjusted by age and total cholesterol.

‡ Analysis considering the median value of the markers.

CIMT: carotid intima-media thickness; OR: odds ratio.

## Discussion

To our knowledge, this is the first study to evaluate inflammatory biomarkers with the presence of CIMT only in individuals considered at low cardiovascular risk, with exclusive use of NRTIs and NNRTIs, and with undetectable HIV-1 RNA viral load in a population of HIV-infected patients.

In the univariate analysis, it was found that inflammatory biomarkers (IFN-γ, IL-1β, and TNF-α) were higher in the non-HIV group. These data are in contrast to the ones by Ross et al.,^2^ who found that TNF-α, hs-CRP, IL-6, and sVCAM-1 were significantly higher in the HIV-infected group. Bethan et al.^11^ also observed higher elevation of IL-6 and C-reactive protein in HIV-positives as compared with controls. Our study excluded patients with detectable viral load, which is a contributing factor to the increase of these markers. In contrast, the studies cited did not use detectable viral load as an exclusion criterion, and it may have been an influencing factor in the discordance of the results. Samples obtained from plasma donors before, during, and after HIV acquisition demonstrated elevations in various cytokines during viral expansion,^12^ and the initiation of ART in chronic infection is associated with a decline in the circulating levels of some cytokines, including IL-1β, IL-6, and TNF-α, possibly by reduction of viral load.^13^ An important limitation of the present study is the fact that the non- HIV group consisted of older individuals as compared with the infected ones. This factor may have contributed to the determination of higher levels of the abovementioned inflammatory markers and total and LDL cholesterol and consequently may have also skewed the results of the association of total and LDL cholesterol with CIMT ≥ 0.61 mm. However, it is worth noting that there is a great divergence in several studies under lipidogram changes in HIV-infected individuals^2,14^. For example, in the study by Ross et al.,^2^ the HIV-infected group had lower mean HDL, but total cholesterol, triglycerides, and LDL were similar between the groups. LDL and triglyceride levels were positively correlated with CIMT. HIV-infected individuals had significantly higher triglyceride values and lower values of total cholesterol, HDL, and LDL as compared with the control group.^14,15^

It has been shown that individuals over 40 years of age presented significantly higher total cholesterol, HDL and LDL levels, and higher mean CIMT, reinforcing that older age is a factor associated with its altered thickness measurement. CIMT means showed no statistically significant difference when compared to individuals with and without HIV. Lorenz et al.^16^ demonstrated higher CIMT mean in the HIV group when compared with the control one. According to Falcão et al.,^17^ patients classified as having medium or high cardiovascular risk based on the Framingham score were 3.7 times more likely to present atherosclerosis than patients considered at low risk. In another study, patients with subclinical atherosclerosis had higher risk score compared to those with normal to mid-normal thickness. For every 10% increase in the FRS, the odds of having an abnormal CIMT tripled.^18^ However, the low number of individuals in the control group indicates another important limitation for our results, which may explain the lack of statistical association of carotid thickness and HIV infection. Thus, in later studies, we would need a larger group of individuals to further investigate this hypothesis.

Higher mean age was associated with higher CIMT in HIV-infected and non-infected individuals. Stratified analysis, when controlling for age and CIMT by the infection status, verified that HIV infection does not interfere in the association, that is, the aforementioned relation is independent of the infection status in the analyzed population. However, a larger sample size would be necessary to give greater statistical power to the analysis, considering the wide confidence interval of the raw odds ratios and Mantel-Haenszel odds.

When evaluating the association between inflammatory markers and CIMT, a significant association with TNF-α was observed in the multivariate analysis, as the increase in IL1-β levels presented a greater chance of atherosclerosis with p-value close to significance. Ssinabulya et al.^18^ found high levels of us-CRP were not associated with CIMT. However, in other studies, higher CIMT was associated with higher levels of IL-2, IL-6, TNF-α, us-CRP, and sVCAM-1.^2,3^ Both our studies and other ones cited have small sample size, so further investigation is needed to more accurately confirm the relationship between inflammatory markers and the occurrence of carotid atherosclerosis. Our result can be explained by careful selection of our patients. Both are attended at a referral centre for HIV treatment, with good medical follow-up. Our sample evaluates an “ideal” patient, who has undetectable viral load for probably a rather long time, since the time of ART is greater than five years in 63% and without previous or current use of protease inhibitors. In addition, the FRS calculation for cardiovascular disease is low. These data point to the importance of patient awareness among health professionals, guiding them and controlling risk factors such as smoking, diabetes, hypertension, and dyslipidaemia.

## Conclusions

HIV-positive individuals with undetectable HIV-1 RNA viral load, at low risk for cardiovascular disease, using NRTI and NNRTI presented similar carotid thickness compared with non-infected people. Inflammatory markers IL-6, hs-CRP, sVCAM-1, and sICAM-1 showed similar levels in the studied groups and IFN-γ, IL-1, and TNF-α had lower levels in the HIV population. Evaluating the association between inflammatory markers and CIMT, in the multivariate analysis, TNF-α and IL1-β were shown to be associated with a greater chance of higher carotid thickness. Our study demonstrates that HIV-positive individuals with undetectable viral load in ART without protease inhibitors and with low cardiovascular risk do not present differences in carotid thickness in relation to uninfected individuals. Control of viral load with NRTIs and NNRTI plus the maintenance of cardiovascular risk parameters under control - such as smoking, diabetes, and dyslipidaemia - possibly result in the patient with HIV having lower risk of occurrence of subclinical atherosclerosis.
